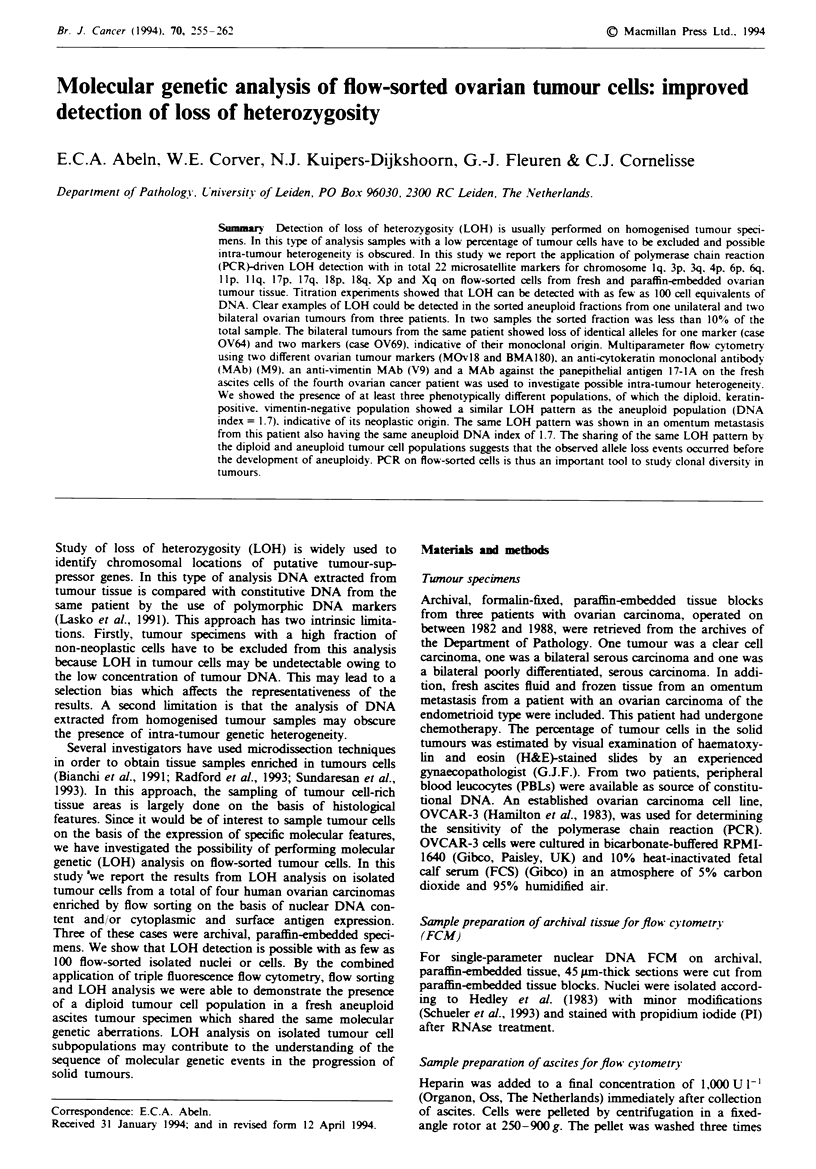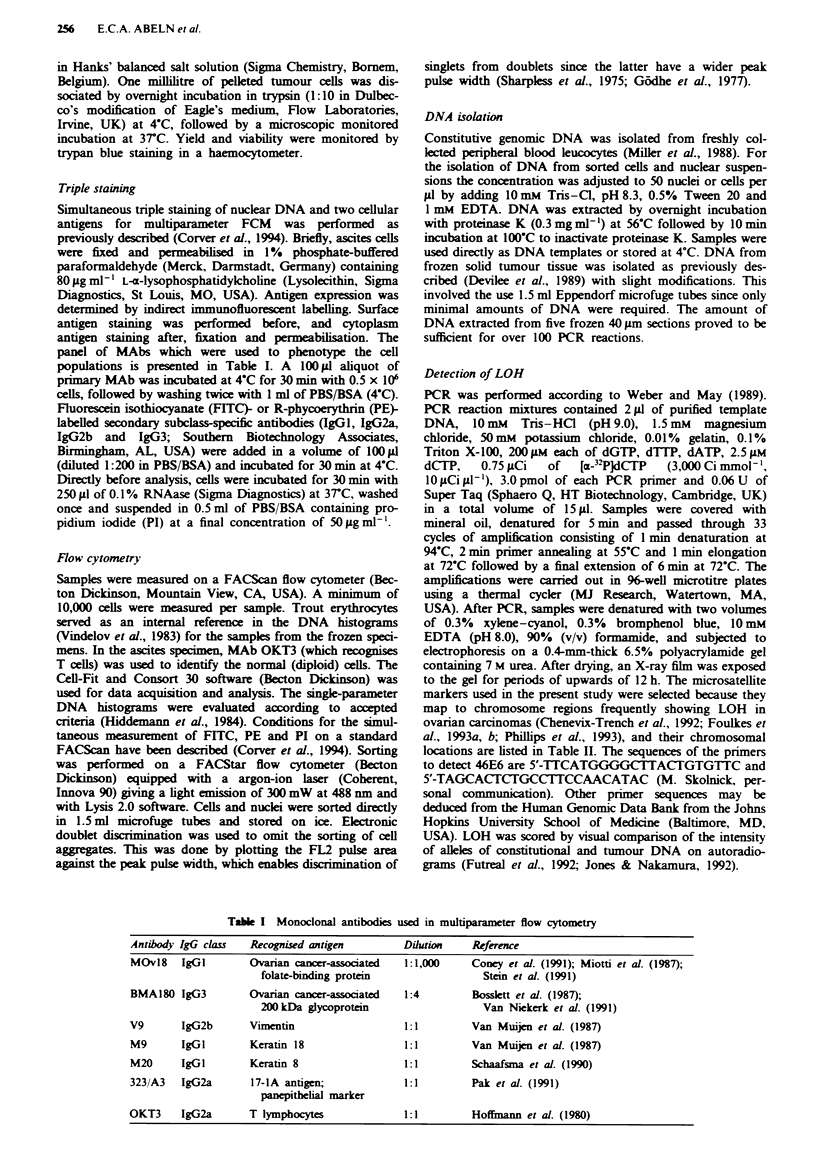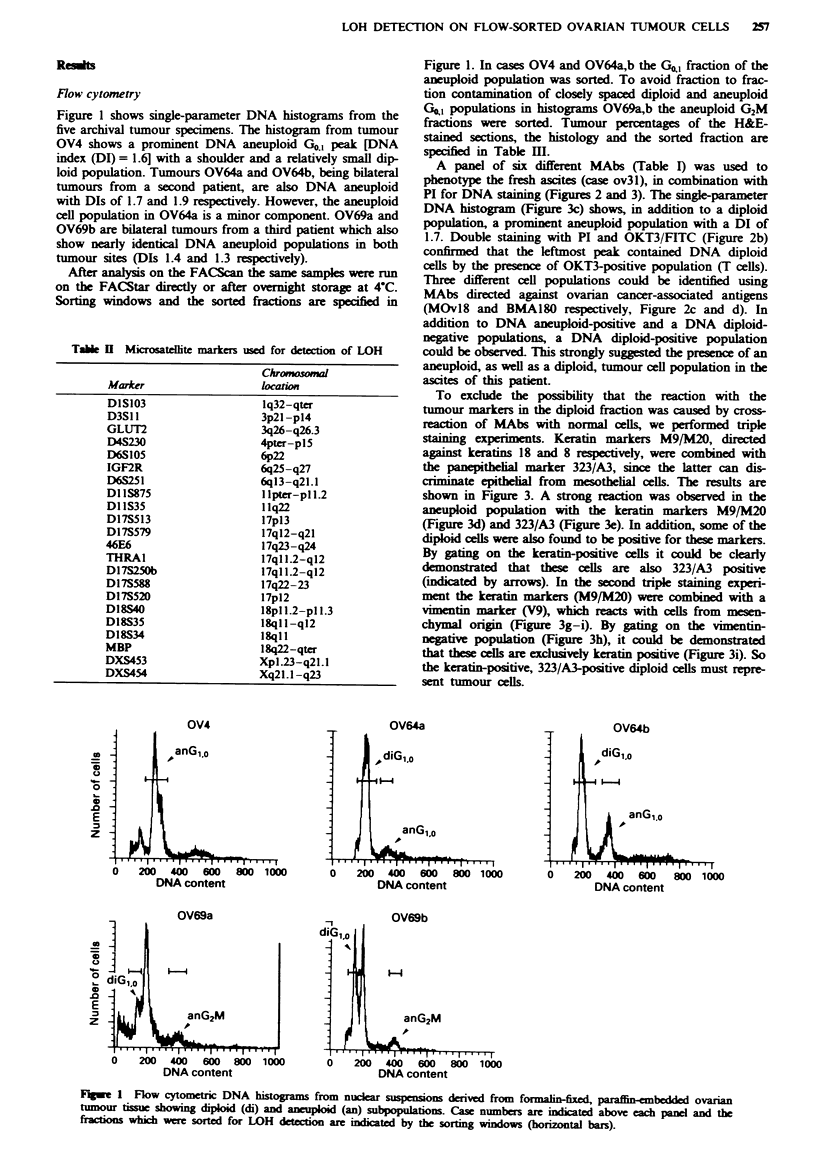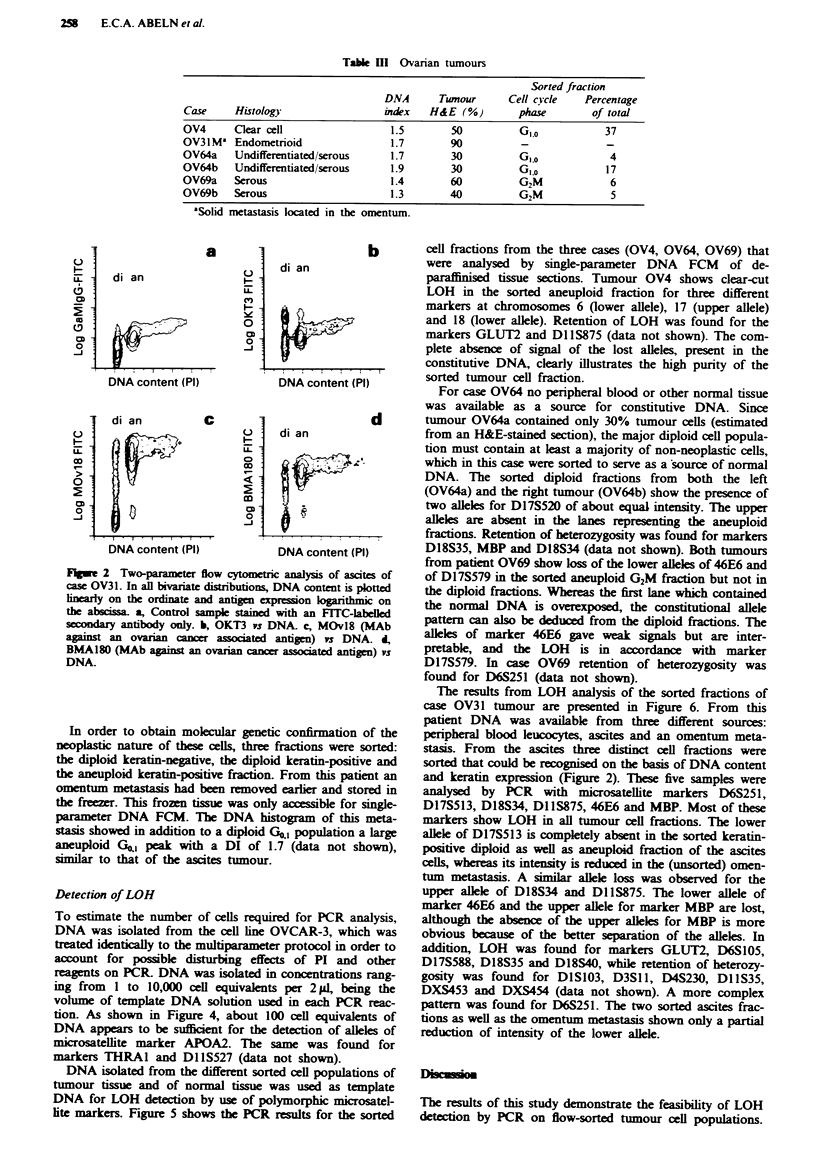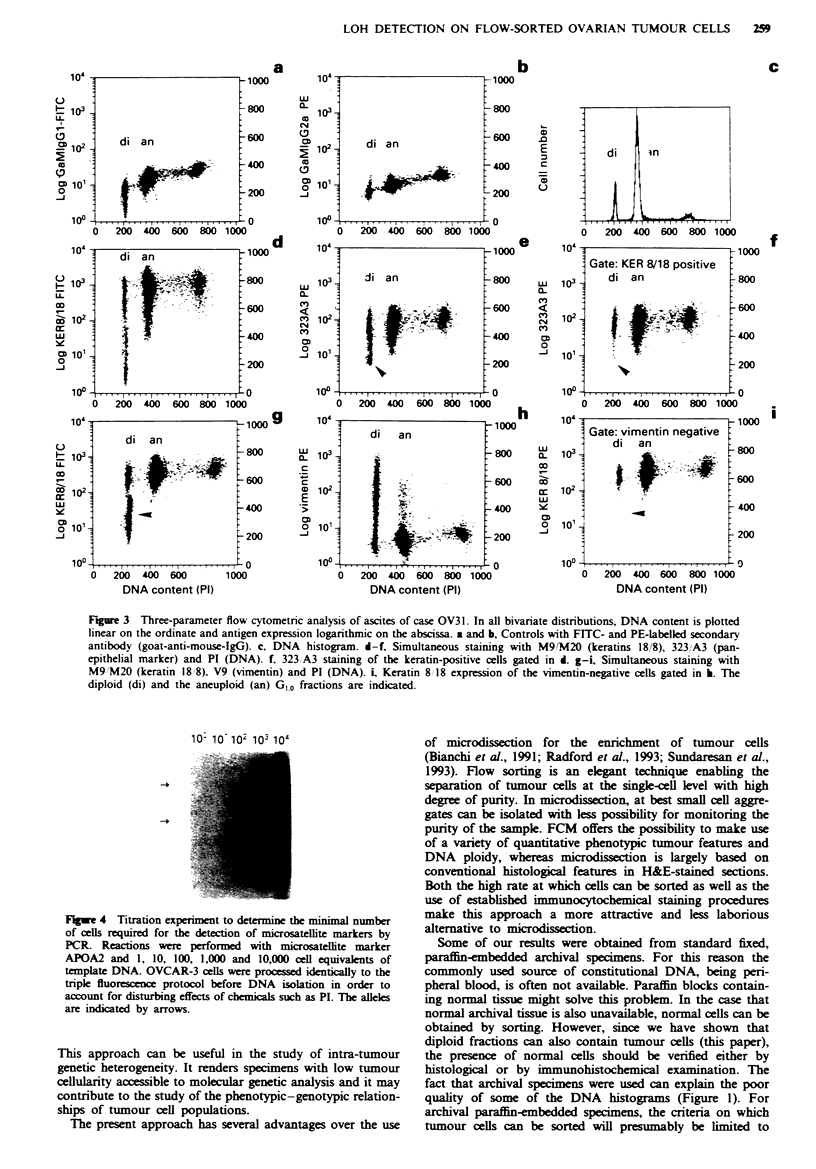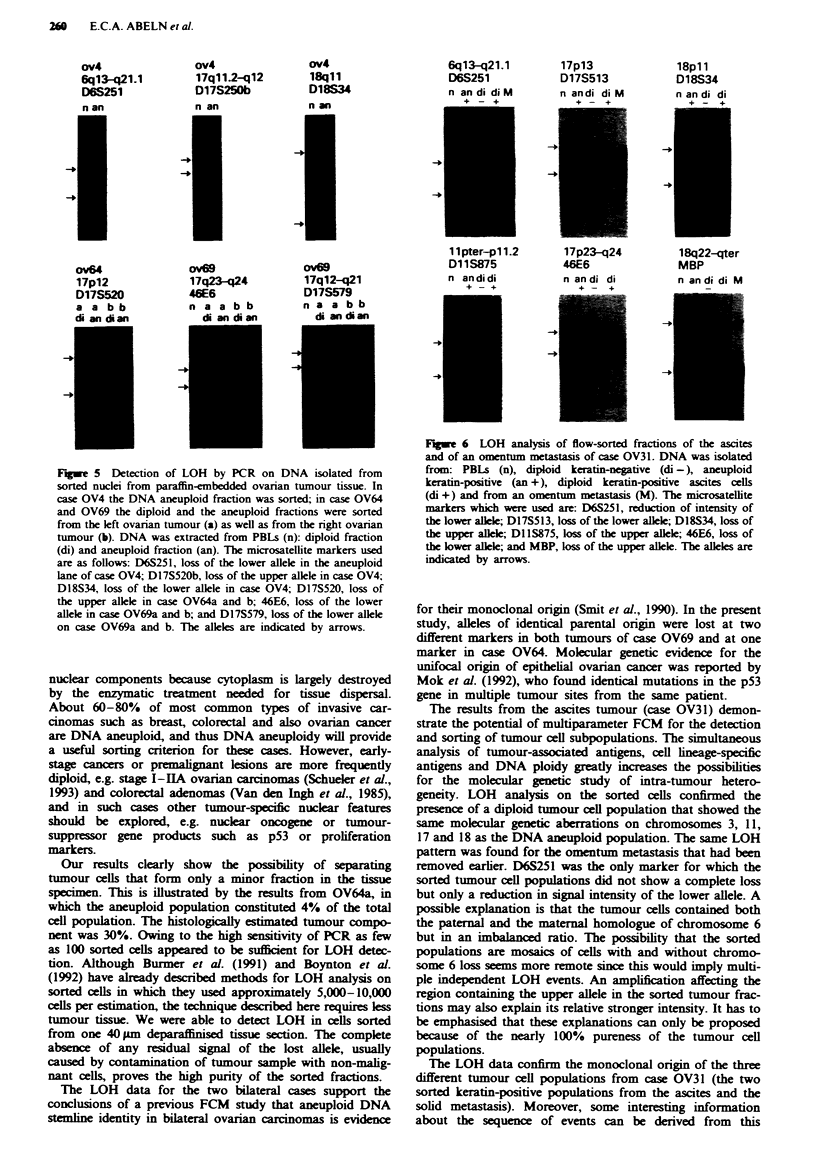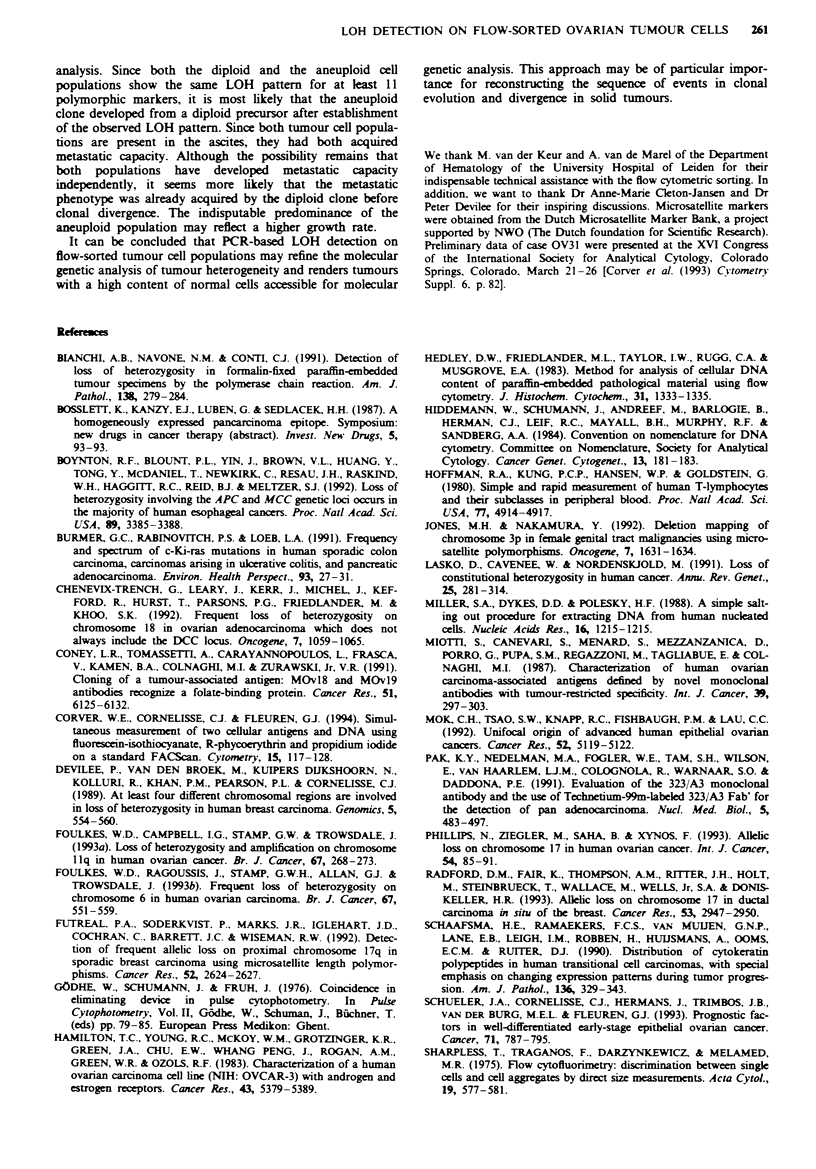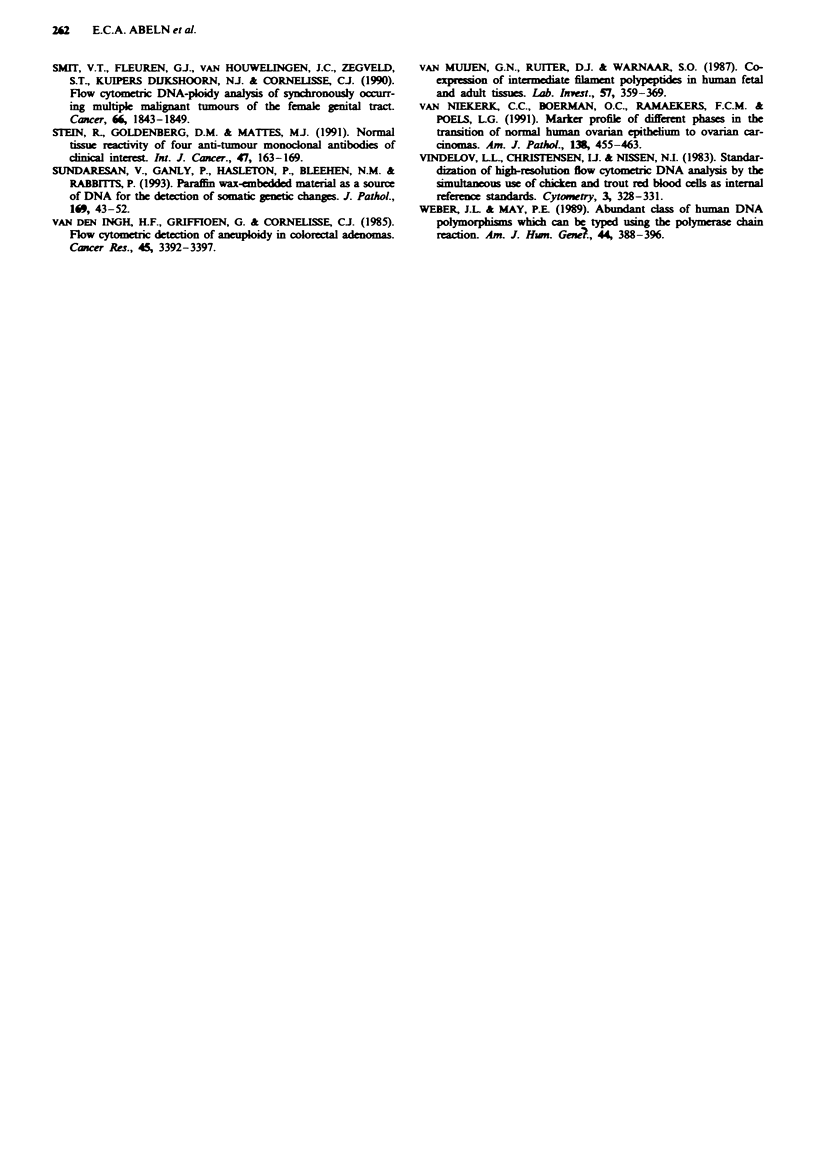# Molecular genetic analysis of flow-sorted ovarian tumour cells: improved detection of loss of heterozygosity.

**DOI:** 10.1038/bjc.1994.289

**Published:** 1994-08

**Authors:** E. C. Abeln, W. E. Corver, N. J. Kuipers-Dijkshoorn, G. J. Fleuren, C. J. Cornelisse

**Affiliations:** Department of Pathology, University of Leiden, The Netherlands.

## Abstract

**Images:**


Br. J. Cancer (1994), 70, 255-262 © Macmillan Press Ltd., 1994

### Molecular genetic analysis of flow-sorted ovarian tumour cells: improved detection of loss of heterozygosity

E.C.A. Abeln, W.E. Corver, N.J. Kuipers-Dijkshoorn, G.-J. Fleuren & C.J. Cornelisse

*Department of Pathology, University of Leiden, PO Box 96030, 2300 RC Leiden, The Netherlands.*

**Summary** Detection of loss of heterozygosity (LOH) is usually performed on homogenised tumour specimens. In this type of analysis samples with a low percentage of tumour cells have to be excluded and possible intra-tumour heterogeneity is obscured. In this study we report the application of polymerase chain reaction (PCR)-driven LOH detection with in total 22 microsatellite markers for chromosome 1q, 3p, 3q, 4p, 6p, 6q, 11p, 11q, 17p, 17q, 18p, 18q, Xp and Xq on flow-sorted cells from fresh and paraffin-embedded ovarian tumour tissue. Titration experiments showed that LOH can be detected with as few as 100 cell equivalents of DNA. Clear examples of LOH could be detected in the sorted aneuploid fractions from one unilateral and two bilateral ovarian tumours from three patients. In two samples the sorted fraction was less than 10% of the total sample. The bilateral tumours from the same patient showed loss of identical alleles for one marker (case OV64) and two markers (case OV69), indicative of their monoclonal origin. Multiparameter flow cytometry using two different ovarian tumour markers (MOv18 and BMA180), an anti-cytokeratin monoclonal antibody (MAb) (M9), an anti-vimentin MAb (V9) and a MAb against the panepithelial antigen 17-1A on the fresh ascites cells of the fourth ovarian cancer patient was used to investigate possible intra-tumour heterogeneity. We showed the presence of at least three phenotypically different populations, of which the diploid, keratin-positive, vimentin-negative population showed a similar LOH pattern as the aneuploid population (DNA index = 1.7), indicative of its neoplastic origin. The same LOH pattern was shown in an omentum metastasis from this patient also having the same aneuploid DNA index of 1.7. The sharing of the same LOH pattern by the diploid and aneuploid tumour cell populations suggests that the observed allele loss events occurred before the development of aneuploidy. PCR on flow-sorted cells is thus an important tool to study clonal diversity in tumours.

Study of loss of heterozygosity (LOH) is widely used to identify chromosomal locations of putative tumour-suppressor genes. In this type of analysis DNA extracted from tumour tissue is compared with constitutive DNA from the same patient by the use of polymorphic DNA markers (Lasko *et al.*, 1991). This approach has two intrinsic limitations. Firstly, tumour specimens with a high fraction of non-neoplastic cells have to be excluded from this analysis because LOH in tumour cells may be undetectable owing to the low concentration of tumour DNA. This may lead to a selection bias which affects the representativeness of the results. A second limitation is that the analysis of DNA extracted from homogenised tumour samples may obscure the presence of intra-tumour genetic heterogeneity.

Several investigators have used microdissection techniques in order to obtain tissue samples enriched in tumours cells (Bianchi *et al.*, 1991; Radford *et al.*, 1993; Sundaresan *et al.*, 1993). In this approach, the sampling of tumour cell-rich tissue areas is largely done on the basis of histological features. Since it would be of interest to sample tumour cells on the basis of the expression of specific molecular features, we have investigated the possibility of performing molecular genetic (LOH) analysis on flow-sorted tumour cells. In this study we report the results from LOH analysis on isolated tumour cells from a total of four human ovarian carcinomas enriched by flow sorting on the basis of nuclear DNA content and/or cytoplasmic and surface antigen expression. Three of these cases were archival, paraffin-embedded specimens. We show that LOH detection is possible with as few as 100 flow-sorted isolated nuclei or cells. By the combined application of triple fluorescence flow cytometry, flow sorting and LOH analysis we were able to demonstrate the presence of a diploid tumour cell population in a fresh aneuploid ascites tumour specimen which shared the same molecular genetic aberrations. LOH analysis on isolated tumour cell subpopulations may contribute to the understanding of the sequence of molecular genetic events in the progression of solid tumours.

Correspondence: E.C.A. Abeln.
Received 31 January 1994; and in revised form 12 April 1994.

### Materials and methods

### *Tumour specimens*

Archival, formalin-fixed, paraffin-embedded tissue blocks from three patients with ovarian carcinoma, operated on between 1982 and 1988, were retrieved from the archives of the Department of Pathology. One tumour was a clear cell carcinoma, one was a bilateral serous carcinoma and one was a bilateral poorly differentiated, serous carcinoma. In addition, fresh ascites fluid and frozen tissue from an omentum metastasis from a patient with an ovarian carcinoma of the endometrioid type were included. This patient had undergone chemotherapy. The percentage of tumour cells in the solid tumours was estimated by visual examination of haematoxylin and eosin (H&E)-stained slides by an experienced gynaecopathologist (G.J.F.). From two patients, peripheral blood leucocytes (PBLs) were available as source of constitutional DNA. An established ovarian carcinoma cell line, OVCAR-3 (Hamilton *et al.*, 1983), was used for determining the sensitivity of the polymerase chain reaction (PCR). OVCAR-3 cells were cultured in bicarbonate-buffered RPMI-1640 (Gibco, Paisley, UK) and 10% heat-inactivated fetal calf serum (FCS) (Gibco) in an atmosphere of 5% carbon dioxide and 95% humidified air.

### *Sample preparation of archival tissue for flow cytometry (FCM)*

For single-parameter nuclear DNA FCM on archival, paraffin-embedded tissue, 45 μm-thick sections were cut from paraffin-embedded tissue blocks. Nuclei were isolated according to Hedley *et al.* (1983) with minor modifications (Schueler *et al.*, 1993) and stained with propidium iodide (PI) after RNAse treatment.

### *Sample preparation of ascites for flow cytometry*

Heparin was added to a final concentration of 1,000 U $l^{-1}$ (Organon, Oss, The Netherlands) immediately after collection of ascites. Cells were pelleted by centrifugation in a fixed-angle rotor at 250-900 *g*. The pellet was washed three times in Hanks' balanced salt solution (Sigma Chemistry, Bornem, Belgium). One millilitre of pelleted tumour cells was dissociated by overnight incubation in trypsin (1:10 in Dulbecco's modification of Eagle's medium, Flow Laboratories, Irvine, UK) at 4°C, followed by a microscopic monitored incubation at 37°C. Yield and viability were monitored by trypan blue staining in a haemocytometer.

*Triple staining*

Simultaneous triple staining of nuclear DNA and two cellular antigens for multiparameter FCM was performed as previously described (Corver *et al.*, 1994). Briefly, ascites cells were fixed and permeabilised in 1% phosphate-buffered paraformaldehyde (Merck, Darmstadt, Germany) containing 80 $\mu g\ ml^{-1}$ L-α-lysophosphatidylcholine (Lysolecithin, Sigma Diagnostics, St Louis, MO, USA). Antigen expression was determined by indirect immunofluorescent labelling. Surface antigen staining was performed before, and cytoplasm antigen staining after, fixation and permeabilisation. The panel of MAbs which were used to phenotype the cell populations is presented in Table I. A 100 µl aliquot of primary MAb was incubated at 4°C for 30 min with $0.5 \times 10^6$ cells, followed by washing twice with 1 ml of PBS/BSA (4°C). Fluorescein isothiocyanate (FITC)- or R-phycoerythrin (PE)-labelled secondary subclass-specific antibodies (IgG1, IgG2a, IgG2b and IgG3; Southern Biotechnology Associates, Birmingham, AL, USA) were added in a volume of 100 µl (diluted 1:200 in PBS/BSA) and incubated for 30 min at 4°C. Directly before analysis, cells were incubated for 30 min with 250 µl of 0.1% RNAase (Sigma Diagnostics) at 37°C, washed once and suspended in 0.5 ml of PBS/BSA containing propidium iodide (PI) at a final concentration of 50 $\mu g\ ml^{-1}$.

*Flow cytometry*

Samples were measured on a FACScan flow cytometer (Becton Dickinson, Mountain View, CA, USA). A minimum of 10,000 cells were measured per sample. Trout erythrocytes served as an internal reference in the DNA histograms (Vindelov *et al.*, 1983) for the samples from the frozen specimens. In the ascites specimen, MAb OKT3 (which recognises T cells) was used to identify the normal (diploid) cells. The Cell-Fit and Consort 30 software (Becton Dickinson) was used for data acquisition and analysis. The single-parameter DNA histograms were evaluated according to accepted criteria (Hiddemann *et al.*, 1984). Conditions for the simultaneous measurement of FITC, PE and PI on a standard FACScan have been described (Corver *et al.*, 1994). Sorting was performed on a FACStar flow cytometer (Becton Dickinson) equipped with a argon-ion laser (Coherent, Innova 90) giving a light emission of 300 mW at 488 nm and with Lysis 2.0 software. Cells and nuclei were sorted directly in 1.5 ml microfuge tubes and stored on ice. Electronic doublet discrimination was used to omit the sorting of cell aggregates. This was done by plotting the FL2 pulse area against the peak pulse width, which enables discrimination of singlets from doublets since the latter have a wider peak pulse width (Sharpless *et al.*, 1975; Gödhe *et al.*, 1977).

*DNA isolation*

Constitutive genomic DNA was isolated from freshly collected peripheral blood leucocytes (Miller *et al.*, 1988). For the isolation of DNA from sorted cells and nuclear suspensions the concentration was adjusted to 50 nuclei or cells per µl by adding 10 mM Tris-Cl, pH 8.3, 0.5% Tween 20 and 1 mM EDTA. DNA was extracted by overnight incubation with proteinase K (0.3 $mg\ ml^{-1}$) at 56°C followed by 10 min incubation at 100°C to inactivate proteinase K. Samples were used directly as DNA templates or stored at 4°C. DNA from frozen solid tumour tissue was isolated as previously described (Devilee *et al.*, 1989) with slight modifications. This involved the use 1.5 ml Eppendorf microfuge tubes since only minimal amounts of DNA were required. The amount of DNA extracted from five frozen 40 µm sections proved to be sufficient for over 100 PCR reactions.

*Detection of LOH*

PCR was performed according to Weber and May (1989). PCR reaction mixtures contained 2 µl of purified template DNA, 10 mM Tris-HCl (pH 9.0), 1.5 mM magnesium chloride, 50 mM potassium chloride, 0.01% gelatin, 0.1% Triton X-100, 200 µM each of dGTP, dTTP, dATP, 2.5 µM dCTP, 0.75 µCi of [α-$^{32}$P]dCTP (3,000 $Ci\ mmol^{-1}$, 10 $\mu Ci\ \mu l^{-1}$), 3.0 pmol of each PCR primer and 0.06 U of Super Taq (Sphaero Q, HT Biotechnology, Cambridge, UK) in a total volume of 15 µl. Samples were covered with mineral oil, denatured for 5 min and passed through 33 cycles of amplification consisting of 1 min denaturation at 94°C, 2 min primer annealing at 55°C and 1 min elongation at 72°C followed by a final extension of 6 min at 72°C. The amplifications were carried out in 96-well microtitre plates using a thermal cycler (MJ Research, Watertown, MA, USA). After PCR, samples were denatured with two volumes of 0.3% xylene-cyanol, 0.3% bromphenol blue, 10 mM EDTA (pH 8.0), 90% (v/v) formamide, and subjected to electrophoresis on a 0.4-mm-thick 6.5% polyacrylamide gel containing 7 M urea. After drying, an X-ray film was exposed to the gel for periods of upwards of 12 h. The microsatellite markers used in the present study were selected because they map to chromosome regions frequently showing LOH in ovarian carcinomas (Chenevix-Trench *et al.*, 1992; Foulkes *et al.*, 1993*a*, *b*; Phillips *et al.*, 1993), and their chromosomal locations are listed in Table II. The sequences of the primers to detect 46E6 are 5'-TTCATGGGGCTTACTGTGTTC and 5'-TAGCACTCTGCCTTCCAACATAC (M. Skolnick, personal communication). Other primer sequences may be deduced from the Human Genomic Data Bank from the Johns Hopkins University School of Medicine (Baltimore, MD, USA). LOH was scored by visual comparison of the intensity of alleles of constitutional and tumour DNA on autoradiograms (Futreal *et al.*, 1992; Jones & Nakamura, 1992).

**Table I** Monoclonal antibodies used in multiparameter flow cytometry

| *Antibody* | *IgG class* | *Recognised antigen* | *Dilution* | *Reference* |
|---|---|---|---|---|
| MOv18 | IgG1 | Ovarian cancer-associated folate-binding protein | 1:1,000 | Coney *et al.* (1991); Miotti *et al.* (1987); Stein *et al.* (1991) |
| BMA180 | IgG3 | Ovarian cancer-associated 200 kDa glycoprotein | 1:4 | Bosslett *et al.* (1987); Van Niekerk *et al.* (1991) |
| V9 | IgG2b | Vimentin | 1:1 | Van Muijen *et al.* (1987) |
| M9 | IgG1 | Keratin 18 | 1:1 | Van Muijen *et al.* (1987) |
| M20 | IgG1 | Keratin 8 | 1:1 | Schaafsma *et al.* (1990) |
| 323/A3 | IgG2a | 17-1A antigen; panepithelial marker | 1:1 | Pak *et al.* (1991) |
| OKT3 | IgG2a | T lymphocytes | 1:1 | Hoffmann *et al.* (1980) |

### Results

### *Flow cytometry*

Figure 1 shows single-parameter DNA histograms from the five archival tumour specimens. The histogram from tumour OV4 shows a prominent DNA aneuploid $G_{0,1}$ peak [DNA index (DI) = 1.6] with a shoulder and a relatively small diploid population. Tumours OV64a and OV64b, being bilateral tumours from a second patient, are also DNA aneuploid with DIs of 1.7 and 1.9 respectively. However, the aneuploid cell population in OV64a is a minor component. OV69a and OV69b are bilateral tumours from a third patient which also show nearly identical DNA aneuploid populations in both tumour sites (DIs 1.4 and 1.3 respectively).

After analysis on the FACScan the same samples were run on the FACStar directly or after overnight storage at 4°C. Sorting windows and the sorted fractions are specified in Figure 1. In cases OV4 and OV64a,b the $G_{0,1}$ fraction of the aneuploid population was sorted. To avoid fraction to fraction contamination of closely spaced diploid and aneuploid $G_{0,1}$ populations in histograms OV69a,b the aneuploid $G_2M$ fractions were sorted. Tumour percentages of the H&E-stained sections, the histology and the sorted fraction are specified in Table III.

**Table II** Microsatellite markers used for detection of LOH

| *Marker* | *Chromosomal location* |
|---|---|
| D1S103 | 1q32-qter |
| D3S11 | 3p21-p14 |
| GLUT2 | 3q26-q26.3 |
| D4S230 | 4pter-p15 |
| D6S105 | 6p22 |
| IGF2R | 6q25-q27 |
| D6S251 | 6q13-q21.1 |
| D11S875 | 11pter-p11.2 |
| D11S35 | 11q22 |
| D17S513 | 17p13 |
| D17S579 | 17q12-q21 |
| 46E6 | 17q23-q24 |
| THRA1 | 17q11.2-q12 |
| D17S250b | 17q11.2-q12 |
| D17S588 | 17q22-23 |
| D17S520 | 17p12 |
| D18S40 | 18p11.2-p11.3 |
| D18S35 | 18q11-q12 |
| D18S34 | 18q11 |
| MBP | 18q22-qter |
| DXS453 | Xp1.23-q21.1 |
| DXS454 | Xq21.1-q23 |

A panel of six different MAbs (Table I) was used to phenotype the fresh ascites (case ov31), in combination with PI for DNA staining (Figures 2 and 3). The single-parameter DNA histogram (Figure 3c) shows, in addition to a diploid population, a prominent aneuploid population with a DI of 1.7. Double staining with PI and OKT3/FITC (Figure 2b) confirmed that the leftmost peak contained DNA diploid cells by the presence of OKT3-positive population (T cells). Three different cell populations could be identified using MAbs directed against ovarian cancer-associated antigens (MOv18 and BMA180 respectively, Figure 2c and d). In addition to DNA aneuploid-positive and a DNA diploid-negative populations, a DNA diploid-positive population could be observed. This strongly suggested the presence of an aneuploid, as well as a diploid, tumour cell population in the ascites of this patient.

To exclude the possibility that the reaction with the tumour markers in the diploid fraction was caused by cross-reaction of MAbs with normal cells, we performed triple staining experiments. Keratin markers M9/M20, directed against keratins 18 and 8 respectively, were combined with the panepithelial marker 323/A3, since the latter can discriminate epithelial from mesothelial cells. The results are shown in Figure 3. A strong reaction was observed in the aneuploid population with the keratin markers M9/M20 (Figure 3d) and 323/A3 (Figure 3e). In addition, some of the diploid cells were also found to be positive for these markers. By gating on the keratin-positive cells it could be clearly demonstrated that these cells are also 323/A3 positive (indicated by arrows). In the second triple staining experiment the keratin markers (M9/M20) were combined with a vimentin marker (V9), which reacts with cells from mesenchymal origin (Figure 3g-i). By gating on the vimentin-negative population (Figure 3h), it could be demonstrated that these cells are exclusively keratin positive (Figure 3i). So the keratin-positive, 323/A3-positive diploid cells must represent tumour cells.

**Figure 1** Flow cytometric DNA histograms from nuclear suspensions derived from formalin-fixed, paraffin-embedded ovarian tumour tissue showing diploid (di) and aneuploid (an) subpopulations. Case numbers are indicated above each panel and the fractions which were sorted for LOH detection are indicated by the sorting windows (horizontal bars).

**Table III** Ovarian tumours

| Case | Histology | DNA index | Tumour H&E (%) | Sorted fraction Cell cycle phase | Percentage of total |
|---|---|---|---|---|---|
| OV4 | Clear cell | 1.5 | 50 | $G_{1,0}$ | 37 |
| OV31M[a] | Endometrioid | 1.7 | 90 | – | – |
| OV64a | Undifferentiated/serous | 1.7 | 30 | $G_{1,0}$ | 4 |
| OV64b | Undifferentiated/serous | 1.9 | 30 | $G_{1,0}$ | 17 |
| OV69a | Serous | 1.4 | 60 | $G_2M$ | 6 |
| OV69b | Serous | 1.3 | 40 | $G_2M$ | 5 |

[a]Solid metastasis located in the omentum.

**Figure 2** Two-parameter flow cytometric analysis of ascites of case OV31. In all bivariate distributions, DNA content is plotted linearly on the ordinate and antigen expression logarithmic on the abscissa. **a**, Control sample stained with an FITC-labelled secondary antibody only. **b**, OKT3 *vs* DNA. **c**, MOv18 (MAb against an ovarian cancer associated antigen) *vs* DNA. **d**, BMA180 (MAb against an ovarian cancer associated antigen) *vs* DNA.

In order to obtain molecular genetic confirmation of the neoplastic nature of these cells, three fractions were sorted: the diploid keratin-negative, the diploid keratin-positive and the aneuploid keratin-positive fraction. From this patient an omentum metastasis had been removed earlier and stored in the freezer. This frozen tissue was only accessible for single-parameter DNA FCM. The DNA histogram of this metastasis showed in addition to a diploid $G_{0,1}$ population a large aneuploid $G_{0,1}$ peak with a DI of 1.7 (data not shown), similar to that of the ascites tumour.

*Detection of LOH*

To estimate the number of cells required for PCR analysis, DNA was isolated from the cell line OVCAR-3, which was treated identically to the multiparameter protocol in order to account for possible disturbing effects of PI and other reagents on PCR. DNA was isolated in concentrations ranging from 1 to 10,000 cell equivalents per 2 µl, being the volume of template DNA solution used in each PCR reaction. As shown in Figure 4, about 100 cell equivalents of DNA appears to be sufficient for the detection of alleles of microsatellite marker APOA2. The same was found for markers THRA1 and D11S527 (data not shown).

DNA isolated from the different sorted cell populations of tumour tissue and of normal tissue was used as template DNA for LOH detection by use of polymorphic microsatellite markers. Figure 5 shows the PCR results for the sorted cell fractions from the three cases (OV4, OV64, OV69) that were analysed by single-parameter DNA FCM of deparaffinised tissue sections. Tumour OV4 shows clear-cut LOH in the sorted aneuploid fraction for three different markers at chromosomes 6 (lower allele), 17 (upper allele) and 18 (lower allele). Retention of LOH was found for the markers GLUT2 and D11S875 (data not shown). The complete absence of signal of the lost alleles, present in the constitutive DNA, clearly illustrates the high purity of the sorted tumour cell fraction.

For case OV64 no peripheral blood or other normal tissue was available as a source for constitutive DNA. Since tumour OV64a contained only 30% tumour cells (estimated from an H&E-stained section), the major diploid cell population must contain at least a majority of non-neoplastic cells, which in this case were sorted to serve as a source of normal DNA. The sorted diploid fractions from both the left (OV64a) and the right tumour (OV64b) show the presence of two alleles for D17S520 of about equal intensity. The upper alleles are absent in the lanes representing the aneuploid fractions. Retention of heterozygosity was found for markers D18S35, MBP and D18S34 (data not shown). Both tumours from patient OV69 show loss of the lower alleles of 46E6 and of D17S579 in the sorted aneuploid $G_2M$ fraction but not in the diploid fractions. Whereas the first lane which contained the normal DNA is overexposed, the constitutional allele pattern can also be deduced from the diploid fractions. The alleles of marker 46E6 gave weak signals but are interpretable, and the LOH is in accordance with marker D17S579. In case OV69 retention of heterozygosity was found for D6S251 (data not shown).

The results from LOH analysis of the sorted fractions of case OV31 tumour are presented in Figure 6. From this patient DNA was available from three different sources: peripheral blood leucocytes, ascites and an omentum metastasis. From the ascites three distinct cell fractions were sorted that could be recognised on the basis of DNA content and keratin expression (Figure 2). These five samples were analysed by PCR with microsatellite markers D6S251, D17S513, D18S34, D11S875, 46E6 and MBP. Most of these markers show LOH in all tumour cell fractions. The lower allele of D17S513 is completely absent in the sorted keratin-positive diploid as well as aneuploid fraction of the ascites cells, whereas its intensity is reduced in the (unsorted) omentum metastasis. A similar allele loss was observed for the upper allele of D18S34 and D11S875. The lower allele of marker 46E6 and the upper allele for marker MBP are lost, although the absence of the upper alleles for MBP is more obvious because of the better separation of the alleles. In addition, LOH was found for markers GLUT2, D6S105, D17S588, D18S35 and D18S40, while retention of heterozygosity was found for D1S103, D3S11, D4S230, D11S35, DXS453 and DXS454 (data not shown). A more complex pattern was found for D6S251. The two sorted ascites fractions as well as the omentum metastasis shown only a partial reduction of intensity of the lower allele.

### Discussion

The results of this study demonstrate the feasibility of LOH detection by PCR on flow-sorted tumour cell populations.

**Figure 3** Three-parameter flow cytometric analysis of ascites of case OV31. In all bivariate distributions, DNA content is plotted linear on the ordinate and antigen expression logarithmic on the abscissa. **a** and **b**, Controls with FITC- and PE-labelled secondary antibody (goat-anti-mouse-IgG). **c**, DNA histogram. **d–f**, Simultaneous staining with M9/M20 (keratins 18/8), 323/A3 (pan-epithelial marker) and PI (DNA). **f**, 323/A3 staining of the keratin-positive cells gated in **d**. **g–i**, Simultaneous staining with M9/M20 (keratin 18/8), V9 (vimentin) and PI (DNA). **i**, Keratin 8/18 expression of the vimentin-negative cells gated in **h**. The diploid (di) and the aneuploid (an) $G_{1,0}$ fractions are indicated.

**Figure 4** Titration experiment to determine the minimal number of cells required for the detection of microsatellite markers by PCR. Reactions were performed with microsatellite marker APOA2 and 1, 10, 100, 1,000 and 10,000 cell equivalents of template DNA. OVCAR-3 cells were processed identically to the triple fluorescence protocol before DNA isolation in order to account for disturbing effects of chemicals such as PI. The alleles are indicated by arrows.

This approach can be useful in the study of intra-tumour genetic heterogeneity. It renders specimens with low tumour cellularity accessible to molecular genetic analysis and it may contribute to the study of the phenotypic–genotypic relationships of tumour cell populations.

The present approach has several advantages over the use of microdissection for the enrichment of tumour cells (Bianchi *et al.*, 1991; Radford *et al.*, 1993; Sundaresan *et al.*, 1993). Flow sorting is an elegant technique enabling the separation of tumour cells at the single-cell level with high degree of purity. In microdissection, at best small cell aggregates can be isolated with less possibility for monitoring the purity of the sample. FCM offers the possibility to make use of a variety of quantitative phenotypic tumour features and DNA ploidy, whereas microdissection is largely based on conventional histological features in H&E-stained sections. Both the high rate at which cells can be sorted as well as the use of established immunocytochemical staining procedures make this approach a more attractive and less laborious alternative to microdissection.

Some of our results were obtained from standard fixed, paraffin-embedded archival specimens. For this reason the commonly used source of constitutional DNA, being peripheral blood, is often not available. Paraffin blocks containing normal tissue might solve this problem. In the case that normal archival tissue is also unavailable, normal cells can be obtained by sorting. However, since we have shown that diploid fractions can also contain tumour cells (this paper), the presence of normal cells should be verified either by histological or by immunohistochemical examination. The fact that archival specimens were used can explain the poor quality of some of the DNA histograms (Figure 1). For archival paraffin-embedded specimens, the criteria on which tumour cells can be sorted will presumably be limited to

**Figure 5** Detection of LOH by PCR on DNA isolated from sorted nuclei from paraffin-embedded ovarian tumour tissue. In case OV4 the DNA aneuploid fraction was sorted; in case OV64 and OV69 the diploid and the aneuploid fractions were sorted from the left ovarian tumour (**a**) as well as from the right ovarian tumour (**b**). DNA was extracted from PBLs (n): diploid fraction (di) and aneuploid fraction (an). The microsatellite markers used are as follows: D6S251, loss of the lower allele in the aneuploid lane of case OV4; D17S520b, loss of the upper allele in case OV4; D18S34, loss of the lower allele in case OV4; D17S520, loss of the upper allele in case OV64a and b; 46E6, loss of the lower allele in case OV69a and b; and D17S579, loss of the lower allele on case OV69a and b. The alleles are indicated by arrows.

**Figure 6** LOH analysis of flow-sorted fractions of the ascites and of an omentum metastasis of case OV31. DNA was isolated from: PBLs (n), diploid keratin-negative (di −), aneuploid keratin-positive (an +), diploid keratin-positive ascites cells (di +) and from an omentum metastasis (M). The microsatellite markers which were used are: D6S251, reduction of intensity of the lower allele; D17S513, loss of the lower allele; D18S34, loss of the upper allele; D11S875, loss of the upper allele; 46E6, loss of the lower allele; and MBP, loss of the upper allele. The alleles are indicated by arrows.

nuclear components because cytoplasm is largely destroyed by the enzymatic treatment needed for tissue dispersal. About 60–80% of most common types of invasive carcinomas such as breast, colorectal and also ovarian cancer are DNA aneuploid, and thus DNA aneuploidy will provide a useful sorting criterion for these cases. However, early-stage cancers or premalignant lesions are more frequently diploid, e.g. stage I–IIA ovarian carcinomas (Schueler *et al.*, 1993) and colorectal adenomas (Van den Ingh *et al.*, 1985), and in such cases other tumour-specific nuclear features should be explored, e.g. nuclear oncogene or tumour-suppressor gene products such as p53 or proliferation markers.

Our results clearly show the possibility of separating tumour cells that form only a minor fraction in the tissue specimen. This is illustrated by the results from OV64a, in which the aneuploid population constituted 4% of the total cell population. The histologically estimated tumour component was 30%. Owing to the high sensitivity of PCR as few as 100 sorted cells appeared to be sufficient for LOH detection. Although Burmer *et al.* (1991) and Boynton *et al.* (1992) have already described methods for LOH analysis on sorted cells in which they used approximately 5,000–10,000 cells per estimation, the technique described here requires less tumour tissue. We were able to detect LOH in cells sorted from one 40 μm deparaffinised tissue section. The complete absence of any residual signal of the lost allele, usually caused by contamination of tumour sample with non-malignant cells, proves the high purity of the sorted fractions.

The LOH data for the two bilateral cases support the conclusions of a previous FCM study that aneuploid DNA stemline identity in bilateral ovarian carcinomas is evidence for their monoclonal origin (Smit *et al.*, 1990). In the present study, alleles of identical parental origin were lost at two different markers in both tumours of case OV69 and at one marker in case OV64. Molecular genetic evidence for the unifocal origin of epithelial ovarian cancer was reported by Mok *et al.* (1992), who found identical mutations in the p53 gene in multiple tumour sites from the same patient.

The results from the ascites tumour (case OV31) demonstrate the potential of multiparameter FCM for the detection and sorting of tumour cell subpopulations. The simultaneous analysis of tumour-associated antigens, cell lineage-specific antigens and DNA ploidy greatly increases the possibilities for the molecular genetic study of intra-tumour heterogeneity. LOH analysis on the sorted cells confirmed the presence of a diploid tumour cell population that showed the same molecular genetic aberrations on chromosomes 3, 11, 17 and 18 as the DNA aneuploid population. The same LOH pattern was found for the omentum metastasis that had been removed earlier. D6S251 was the only marker for which the sorted tumour cell populations did not show a complete loss but only a reduction in signal intensity of the lower allele. A possible explanation is that the tumour cells contained both the paternal and the maternal homologue of chromosome 6 but in an imbalanced ratio. The possibility that the sorted populations are mosaics of cells with and without chromosome 6 loss seems more remote since this would imply multiple independent LOH events. An amplification affecting the region containing the upper allele in the sorted tumour fractions may also explain its relative stronger intensity. It has to be emphasised that these explanations can only be proposed because of the nearly 100% pureness of the tumour cell populations.

The LOH data confirm the monoclonal origin of the three different tumour cell populations from case OV31 (the two sorted keratin-positive populations from the ascites and the solid metastasis). Moreover, some interesting information about the sequence of events can be derived from this analysis. Since both the diploid and the aneuploid cell populations show the same LOH pattern for at least 11 polymorphic markers, it is most likely that the aneuploid clone developed from a diploid precursor after establishment of the observed LOH pattern. Since both tumour cell populations are present in the ascites, they had both acquired metastatic capacity. Although the possibility remains that both populations have developed metastatic capacity independently, it seems more likely that the metastatic phenotype was already acquired by the diploid clone before clonal divergence. The indisputable predominance of the aneuploid population may reflect a higher growth rate.

It can be concluded that PCR-based LOH detection on flow-sorted tumour cell populations may refine the molecular genetic analysis of tumour heterogeneity and renders tumours with a high content of normal cells accessible for molecular genetic analysis. This approach may be of particular importance for reconstructing the sequence of events in clonal evolution and divergence in solid tumours.

We thank M. van der Keur and A. van de Marel of the Department of Hematology of the University Hospital of Leiden for their indispensable technical assistance with the flow cytometric sorting. In addition, we want to thank Dr Anne-Marie Cleton-Jansen and Dr Peter Devilee for their inspiring discussions. Microsatellite markers were obtained from the Dutch Microsatellite Marker Bank, a project supported by NWO (The Dutch foundation for Scientific Research). Preliminary data of case OV31 were presented at the XVI Congress of the International Society for Analytical Cytology, Colorado Springs, Colorado, March 21-26 [Corver *et al.* (1993) *Cytometry* Suppl. 6, p. 82].

### References

BIANCHI, A.B., NAVONE, N.M. & CONTI, C.J. (1991). Detection of loss of heterozygosity in formalin-fixed paraffin-embedded tumour specimens by the polymerase chain reaction. *Am. J. Pathol.*, **138**, 279-284.

BOSSLETT, K., KANZY, E.J., LUBEN, G. & SEDLACEK, H.H. (1987). A homogeneously expressed pancarcinoma epitope. Symposium: new drugs in cancer therapy (abstract). *Invest. New Drugs*, **5**, 93-93.

BOYNTON, R.F., BLOUNT, P.L., YIN, J., BROWN, V.L., HUANG, Y., TONG, Y., MCDANIEL, T., NEWKIRK, C., RESAU, J.H., RASKIND, W.H., HAGGITT, R.C., REID, B.J. & MELTZER, S.J. (1992). Loss of heterozygosity involving the *APC* and *MCC* genetic loci occurs in the majority of human esophageal cancers. *Proc. Natl Acad. Sci. USA*, **89**, 3385-3388.

BURMER, G.C., RABINOVITCH, P.S. & LOEB, L.A. (1991). Frequency and spectrum of c-Ki-ras mutations in human sporadic colon carcinoma, carcinomas arising in ulcerative colitis, and pancreatic adenocarcinoma. *Environ. Health Perspect.*, **93**, 27-31.

CHENEVIX-TRENCH, G., LEARY, J., KERR, J., MICHEL, J., KEFFORD, R., HURST, T., PARSONS, P.G., FRIEDLANDER, M. & KHOO, S.K. (1992). Frequent loss of heterozygosity on chromosome 18 in ovarian adenocarcinoma which does not always include the DCC locus. *Oncogene*, **7**, 1059-1065.

CONEY, L.R., TOMASSETTI, A., CARAYANNOPOULOS, L., FRASCA, V., KAMEN, B.A., COLNAGHI, M.I. & ZURAWSKI, Jr, V.R. (1991). Cloning of a tumour-associated antigen: MOv18 and MOv19 antibodies recognize a folate-binding protein. *Cancer Res.*, **51**, 6125-6132.

CORVER, W.E., CORNELISSE, C.J. & FLEUREN, G.J. (1994). Simultaneous measurement of two cellular antigens and DNA using fluorescein-isothiocyanate, R-phycoerythrin and propidium iodide on a standard FACScan. *Cytometry*, **15**, 117-128.

DEVILEE, P., VAN DEN BROEK, M., KUIPERS DIJKSHOORN, N., KOLLURI, R., KHAN, P.M., PEARSON, P.L. & CORNELISSE, C.J. (1989). At least four different chromosomal regions are involved in loss of heterozygosity in human breast carcinoma. *Genomics*, **5**, 554-560.

FOULKES, W.D., CAMPBELL, I.G., STAMP, G.W. & TROWSDALE, J. (1993*a*). Loss of heterozygosity and amplification on chromosome 11q in human ovarian cancer. *Br. J. Cancer*, **67**, 268-273.

FOULKES, W.D., RAGOUSSIS, J., STAMP, G.W.H., ALLAN, G.J. & TROWSDALE, J. (1993*b*). Frequent loss of heterozygosity on chromosome 6 in human ovarian carcinoma. *Br. J. Cancer*, **67**, 551-559.

FUTREAL, P.A., SODERKVIST, P., MARKS, J.R., IGLEHART, J.D., COCHRAN, C., BARRETT, J.C. & WISEMAN, R.W. (1992). Detection of frequent allelic loss on proximal chromosome 17q in sporadic breast carcinoma using microsatellite length polymorphisms. *Cancer Res.*, **52**, 2624-2627.

GÖDHE, W., SCHUMANN, J. & FRUH, J. (1976). Coincidence in eliminating device in pulse cytophotometry. In *Pulse Cytophotometry*, Vol. II, Gödhe, W., Schuman, J., Büchner, T. (eds) pp. 79-85. European Press Medikon: Ghent.

HAMILTON, T.C., YOUNG, R.C., MCKOY, W.M., GROTZINGER, K.R., GREEN, J.A., CHU, E.W., WHANG PENG, J., ROGAN, A.M., GREEN, W.R. & OZOLS, R.F. (1983). Characterization of a human ovarian carcinoma cell line (NIH: OVCAR-3) with androgen and estrogen receptors. *Cancer Res.*, **43**, 5379-5389.

HEDLEY, D.W., FRIEDLANDER, M.L., TAYLOR, I.W., RUGG, C.A. & MUSGROVE, E.A. (1983). Method for analysis of cellular DNA content of paraffin-embedded pathological material using flow cytometry. *J. Histochem. Cytochem.*, **31**, 1333-1335.

HIDDEMANN, W., SCHUMANN, J., ANDREEF, M., BARLOGIE, B., HERMAN, C.J., LEIF, R.C., MAYALL, B.H., MURPHY, R.F. & SANDBERG, A.A. (1984). Convention on nomenclature for DNA cytometry. Committee on Nomenclature, Society for Analytical Cytology. *Cancer Genet. Cytogenet.*, **13**, 181-183.

HOFFMAN, R.A., KUNG, P.C.P., HANSEN, W.P. & GOLDSTEIN, G. (1980). Simple and rapid measurement of human T-lymphocytes and their subclasses in peripheral blood. *Proc. Natl Acad. Sci. USA*, **77**, 4914-4917.

JONES, M.H. & NAKAMURA, Y. (1992). Deletion mapping of chromosome 3p in female genital tract malignancies using microsatellite polymorphisms. *Oncogene*, **7**, 1631-1634.

LASKO, D., CAVENEE, W. & NORDENSKJOLD, M. (1991). Loss of constitutional heterozygosity in human cancer. *Annu. Rev. Genet.*, **25**, 281-314.

MILLER, S.A., DYKES, D.D. & POLESKY, H.F. (1988). A simple salting out procedure for extracting DNA from human nucleated cells. *Nucleic Acids Res.*, **16**, 1215-1215.

MIOTTI, S., CANEVARI, S., MENARD, S., MEZZANZANICA, D., PORRO, G., PUPA, S.M., REGAZZONI, M., TAGLIABUE, E. & COLNAGHI, M.I. (1987). Characterization of human ovarian carcinoma-associated antigens defined by novel monoclonal antibodies with tumour-restricted specificity. *Int. J. Cancer*, **39**, 297-303.

MOK, C.H., TSAO, S.W., KNAPP, R.C., FISHBAUGH, P.M. & LAU, C.C. (1992). Unifocal origin of advanced human epithelial ovarian cancers. *Cancer Res.*, **52**, 5119-5122.

PAK, K.Y., NEDELMAN, M.A., FOGLER, W.E., TAM, S.H., WILSON, E., VAN HAARLEM, L.J.M., COLOGNOLA, R., WARNAAR, S.O. & DADDONA, P.E. (1991). Evaluation of the 323/A3 monoclonal antibody and the use of Technetium-99m-labeled 323/A3 Fab' for the detection of pan adenocarcinoma. *Nucl. Med. Biol.*, **5**, 483-497.

PHILLIPS, N., ZIEGLER, M., SAHA, B. & XYNOS, F. (1993). Allelic loss on chromosome 17 in human ovarian cancer. *Int. J. Cancer*, **54**, 85-91.

RADFORD, D.M., FAIR, K., THOMPSON, A.M., RITTER, J.H., HOLT, M., STEINBRUECK, T., WALLACE, M., WELLS, Jr, S.A. & DONIS-KELLER, H.R. (1993). Allelic loss on chromosome 17 in ductal carcinoma *in situ* of the breast. *Cancer Res.*, **53**, 2947-2950.

SCHAAFSMA, H.E., RAMAEKERS, F.C.S., VAN MUIJEN, G.N.P., LANE, E.B., LEIGH, I.M., ROBBEN, H., HUIJSMANS, A., OOMS, E.C.M. & RUITER, D.J. (1990). Distribution of cytokeratin polypeptides in human transitional cell carcinomas, with special emphasis on changing expression patterns during tumor progression. *Am. J. Pathol.*, **136**, 329-343.

SCHUELER, J.A., CORNELISSE, C.J., HERMANS, J., TRIMBOS, J.B., VAN DER BURG, M.E.L. & FLEUREN, G.J. (1993). Prognostic factors in well-differentiated early-stage epithelial ovarian cancer. *Cancer*, **71**, 787-795.

SHARPLESS, T., TRAGANOS, F., DARZYNKEWICZ, & MELAMED, M.R. (1975). Flow cytofluorimetry: discrimination between single cells and cell aggregates by direct size measurements. *Acta Cytol.*, **19**, 577-581.

SMIT, V.T., FLEUREN, G.J., VAN HOUWELINGEN, J.C., ZEGVELD, S.T., KUIPERS DIJKSHOORN, N.J. & CORNELISSE, C.J. (1990). Flow cytometric DNA-ploidy analysis of synchronously occurring multiple malignant tumours of the female genital tract. *Cancer*, **66**, 1843–1849.

STEIN, R., GOLDENBERG, D.M. & MATTES, M.J. (1991). Normal tissue reactivity of four anti-tumour monoclonal antibodies of clinical interest. *Int. J. Cancer.*, **47**, 163–169.

SUNDARESAN, V., GANLY, P., HASLETON, P., BLEEHEN, N.M. & RABBITTS, P. (1993). Paraffin wax-embedded material as a source of DNA for the detection of somatic genetic changes. *J. Pathol.*, **169**, 43–52.

VAN DEN INGH, H.F., GRIFFIOEN, G. & CORNELISSE, C.J. (1985). Flow cytometric detection of aneuploidy in colorectal adenomas. *Cancer Res.*, **45**, 3392–3397.

VAN MUIJEN, G.N., RUITER, D.J. & WARNAAR, S.O. (1987). Co-expression of intermediate filament polypeptides in human fetal and adult tissues. *Lab. Invest.*, **57**, 359–369.

VAN NIEKERK, C.C., BOERMAN, O.C., RAMAEKERS, F.C.M. & POELS, L.G. (1991). Marker profile of different phases in the transition of normal human ovarian epithelium to ovarian carcinomas. *Am. J. Pathol.*, **138**, 455–463.

VINDELOV, L.L., CHRISTENSEN, I.J. & NISSEN, N.I. (1983). Standardization of high-resolution flow cytometric DNA analysis by the simultaneous use of chicken and trout red blood cells as internal reference standards. *Cytometry*, **3**, 328–331.

WEBER, J.L. & MAY, P.E. (1989). Abundant class of human DNA polymorphisms which can be typed using the polymerase chain reaction. *Am. J. Hum. Genet.*, **44**, 388–396.